# Mechanism of First Proton‐Coupled Electron Transfer of Water Oxidation at the ${\mathrm{BiVO}}_{4}$–Water Interface

**DOI:** 10.1002/anie.202507071

**Published:** 2025-05-24

**Authors:** Yong‐Bin Zhuang, Alfredo Pasquarello

**Affiliations:** ^1^ Chaire de Simulation à l'Echelle Atomique (CSEA) Ecole Polytechnique Fédérale de Lausanne (EPFL) Lausanne CH‐1015 Switzerland

**Keywords:** ${\mathrm{BiVO}}_{4}$–water interfaces, Machine learning potentials, Oxygen evolution reaction

## Abstract

The oxygen evolution reaction (OER) at the ${\mathrm{BiVO}}_{4}$–water interface is considered to be the bottleneck of the overall water splitting at this aqueous interface. To provide insight into the mechanism of this reaction, the focus is set on the first proton‐coupled electron transfer (PCET). The free‐energy surface of this first step is obtained by combining on‐the‐fly probability‐enhanced sampling and machine learning potentials at the hybrid functional level of accuracy. Our study reveals that proton transfer precedes electron transfer and determines the reaction barrier, consistent with kinetic‐isotope‐effect experiments. The calculated reaction barrier amounts to 0.66±0.09 eV. The proton moves from the adsorbed water molecule to a surface O atom through a direct transfer mechanism. The hole hopping to the resulting hydroxide occurs via the mediation of a surface Bi atom. The presented framework can be generally applied to other PCET steps and other oxides, thus opening the door to a comprehensive investigation of the OER mechanism at oxide–water interfaces.

## Graphical abstract

The free‐energy surface for the first step of the oxygen evolution reaction at the ${\mathrm{BiOV}}_{4}$–water interface is obtained through machine‐learning potentials at the hybrid‐functional level of accuracy. This work reveals that the proton transfer is the rate‐limiting step, consistent with kinetic‐isotope‐effect experiments.

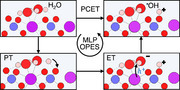

## Introduction

Photocatalytic water splitting was discovered 50 years ago^[^ ^1^ ^]^ and still gathers great attention for its capability of producing clean energy based on hydrogen production. However, the current solar‐to‐hydrogen efficiencies^[^ ^2^ ^]^ of photocatalysts are too low for commercial applications. The oxygen evolution reaction (OER) has been identified as the bottleneck of the water‐splitting process.^[^ ^3^, ^4^ ^]^ This provides a motivation for revealing the underlying atomistic mechanisms through their thermodynamics and kinetics. In this way, we can identify rate‐limiting steps and provide guidance for improving the efficiency of photocatalysts.^[^ ^5^, ^6^ ^]^ Whereas, the thermodynamics has been extensively investigated for promising photoanodes, such as ${\mathrm{TiO}}_{2}$ ^[^ ^7^, ^8^, ^9^ ^]^ and ${\mathrm{BiVO}}_{4}$,^[^ ^10^, ^11^ ^]^ studies of the kinetics^[^ ^12^, ^13^, ^14^, ^15^, ^16^, ^17^ ^]^ have remained scarce as a result of the complex electronic structure and of the water dynamics at the oxide–water interface. In fact, the accurate description of holes either in oxides^[^ ^8^ ^]^ or in water^[^ ^18^ ^]^ requires high‐level electronic‐structure methods, such as hybrid functionals, to capture their polaronic nature. Additionally, it is necessary to account for the water dynamics and for the relatively high barriers of the OER reaction, which can reach values as high as 0.8 eV at typical transition‐metal oxide interfaces.^[^ ^13^, ^14^, ^15^, ^17^, ^19^ ^]^ This requires proper sampling techniques for estimating the free‐energy surface (FES). However, it is a great challenge to converge free‐energy calculations on the time scales, accessible to high‐level electronic‐structure methods.

To address this challenge, simplified schemes have been devised to study the kinetics of the OER. Chen et al. conducted constrained geometry optimization^[^ ^17^, ^19^ ^]^ and Lyu et al. carried out a nudged‐elastic‐band (NEB) simulation^[^ ^15^ ^]^ to obtain potential energy profiles along hole transfer pathways for water oxidation. These approaches neglect the water dynamics and heavily rely on the choices of initial‐state and final‐state configurations. Another scheme combines the QM/MM method and umbrella sampling to construct free‐energy profiles for the OER,^[^ ^14^ ^]^ but suffers from limited sampling, as can be inferred from the dependence on the adopted initial state. In yet another method, the kinetics is characterized through a model based on Marcus' theory with parameters extracted from short ab initio molecular dynamics simulations.^[^ ^16^ ^]^ Overall, the aforementioned schemes all involve approximations to some extent. Ideally, one would like to use a sampling scheme that directly gives the free‐energy surface at the hybrid‐functional level of accuracy.

Machine learning potentials offer the possibility for simulating oxide–water interfaces at the hybrid‐functional level of accuracy at an advantageous computational cost. Over the past decade, this technique has been successfully applied to investigating proton transfer events at aqueous interfaces of oxide materials, including ZnO,^[^ ^20^ ^]^ ${\mathrm{TiO}}_{2}$,^[^ ^21^ ^]^ ${\mathrm{CeO}}_{2}$,^[^ ^22^ ^]^ and ${\mathrm{SnO}}_{2}$.^[^ ^23^ ^]^ Specifically, machine learning potentials allow one to drastically extend simulation time scales making free‐energy profiles of proton reactions accessible. Furthermore, the effectiveness of such potentials has also been demonstrated for describing charge‐transfer events in aqueous solutions.^[^ ^24^, ^25^ ^]^ Therefore, it appears justified to make use of machine learning potentials in the study of the oxygen evolution reaction, in which proton and electron transfer processes occur in a coupled fashion.

Among promising photoanode materials for water splitting in photoelectrochemical cells, ${\mathrm{BiVO}}_{4}$ shows a particularly suitable band gap for visible light adsorption.^[^ ^26^, ^27^, ^28^, ^29^ ^]^ Additionally, ${\mathrm{BiVO}}_{4}$ is thermodynamically active due to convenient band alignment for water oxidation.^[^ ^10^, ^11^, ^30^ ^]^ However, the kinetics of the OER reaction are sluggish as evidenced by experiment.^[^ ^30^ ^]^ For this reason, the OER mechanism at the ${\mathrm{BiVO}}_{4}$–water interface has attracted great attention in experimental investigations.^[^ ^31^, ^32^, ^33^ ^]^ Particularly, kinetic‐isotope‐effect experiments^[^ ^34^, ^35^ ^]^ show that proton transfer is involved in the rate‐limiting step. At variance, theoretical studies of the kinetics^[^ ^15^ ^]^ at this interface have been rare and indicate that electron transfer determines the barrier of the first PCET step. Such a conflicting description motivates further investigation.

In this work, we study the kinetics of the first step of the oxygen evolution reaction at the ${\mathrm{BiVO}}_{4}$–water interface through molecular dynamics simulations. To achieve long time scales combined with high accuracy, we make use of machine learning potentials trained on datasets calculated at the hybrid‐functional level of theory. With these machine learning potentials, we perform on‐the‐fly probability‐enhanced sampling (OPES) method^[^ ^36^ ^]^ to obtain the free‐energy surface of the first proton‐coupled electron transfer (PCET) reaction. We infer the reaction barrier from the minimum energy path and the atomistic mechanism from a probability analysis associated with suitably chosen collective variables. Our work demonstrates the invaluable insight into the mechanism of oxygen evolution reaction that can be acquired through the combination of machine learning potentials and enhanced‐sampling techniques.

## Results

### Free‐Energy Surfaces

**Figure 1:**
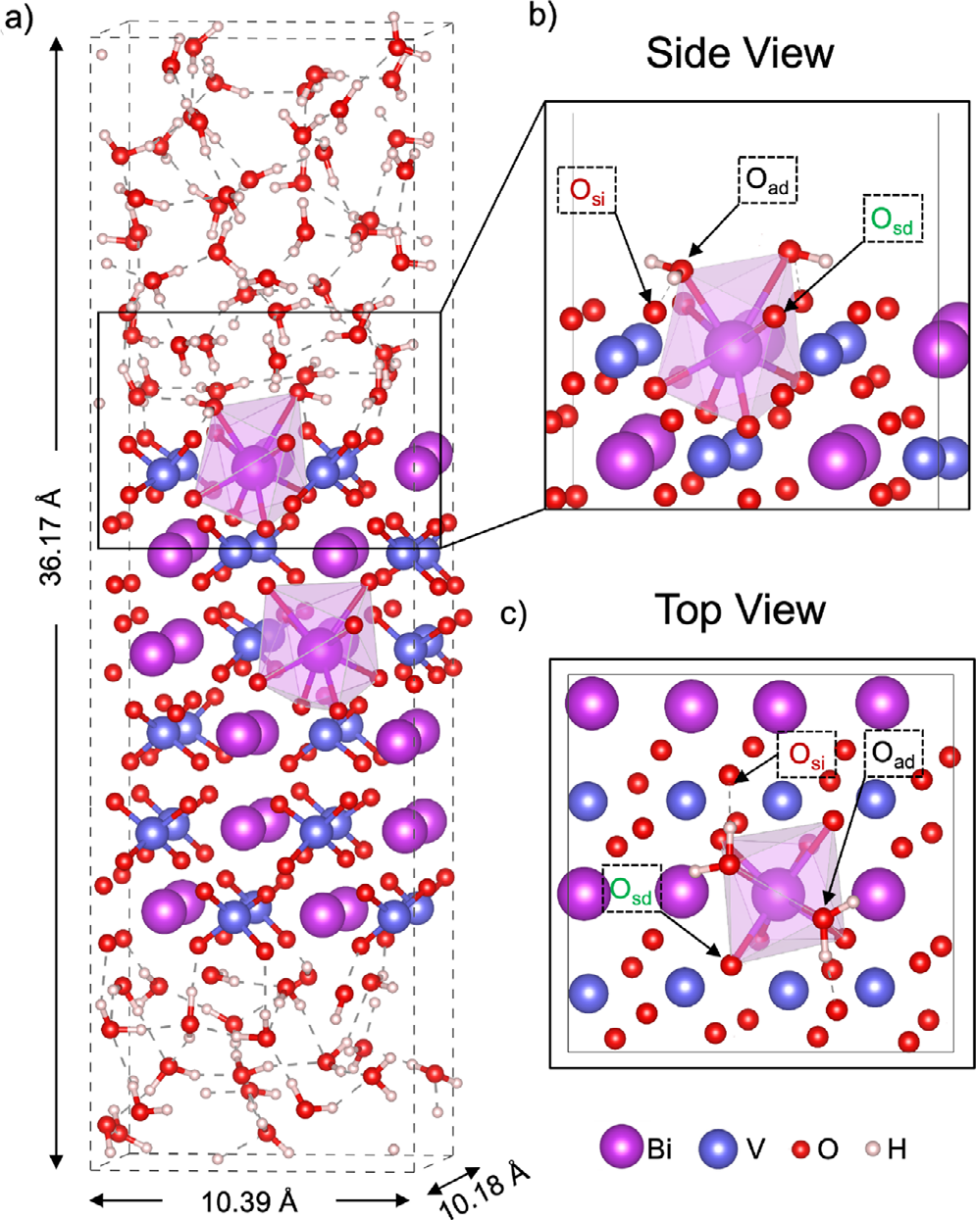
a) Atomistic model of the ${\mathrm{BiVO}}_{4}$–water interface containing 360 atoms. The Bi─O bonds and polyhedra of two selected Bi atoms are visualized. Local b) side and c) top views of the interface model. Irrelevant water molecules and V─O bonds are omitted for clarity. Distinct kinds of oxygen atoms are marked: O atoms belonging to an adsorbed water molecule ($O_{\mathrm{ad}}$), O atoms at the ${\mathrm{BiVO}}_{4}$ surface and directly connected to the selected Bi atom ($O_{\mathrm{sd}}$), and O atoms at the ${\mathrm{BiVO}}_{4}$ surface but not directly connected to the selected Bi atom ($O_{\mathrm{si}}$).

To model the ${\mathrm{BiVO}}_{4}$(010)–water interface, we construct an orthorhombic supercell comprising a ${\mathrm{BiVO}}_{4}$(010) slab of 144 atoms and 72 water molecules. The supercell dimensions are $L_{x}=10.39$, $L_{y}=10.18$, and $L_{z}=$ 36.17 Å (Figure 1a), consistent with previous publications.^[^ ^11^, ^15^ ^]^ We observe that long‐time simulations lead to the spontaneous adsorption of two solvent water molecules per surface Bi site instead of one (cf. Figure 1b, see also Figure S1). In this way, the surface Bi atoms recover the eightfold coordination occurring in the bulk (Figure S2). To avoid this adsorption results in a lower density in the bulk water layer, we include 16 additional water molecules in the supercell. This results in the correct water density in the bulk of the water layer (see Figure S3). All the adsorbed water molecules are in molecular form because $pK_{a}$ of a protonated surface oxygen site is lower than that of adsorbed water by 5–9 units,^[^ ^37^, ^38^ ^]^ which does not allow for water dissociation.

Following ref. [15], we first perform an NEB calculation to obtain the energy profile of the first PCET step of water oxidation as described in the Supporting Information. The NEB reaction barrier is determined to be 1.34 eV (Figure S4). Focusing on a given surface Bi site, the hole polaron is initially mostly located on a surface O atom forming a direct bond with the Bi atom ($O_{\mathrm{sd}}$) and is then gradually transferred to the O atom of an adsorbed water molecule ($O_{\mathrm{ad}}$) bonded to the same Bi atom. This process can be well described through two collective variables (CVs). The first one is the distance between $O_{\mathrm{sd}}$ and $O_{\mathrm{ad}}$, d($O_{\mathrm{sd}}$‐$O_{\mathrm{ad}}$), and is sensitive to electron transfer. Indeed, during the hole transfer process, the d($O_{\mathrm{sd}}$‐$O_{\mathrm{ad}}$) first decreases and then increases, reaching its minimal value at the transition state (Figure S5). The proton transfer is best captured by the number of hydrogen atoms coordinated to $O_{\mathrm{ad}}$, CN($O_{\mathrm{ad}}$‐H). Upon the proton release by the adsorbed water molecule occurring after the transition state, this coordination number shows a sharp drop from 2 to 1 (Figure S5). We remark that in the NEB calculation, the water molecules are allowed to relax but the water dynamics are neglected. Under such constrained conditions, the transition state has been found to depend on the initial‐state and final‐state configurations.^[^ ^17^ ^]^ It is therefore essential to determine the free‐energy profile going beyond these limiting conditions.

To account for the water dynamics, we utilize on‐the‐fly probability enhanced sampling (OPES)^[^ ^36^ ^]^ in conjunction with machine learning potentials. In OPES, it is necessary to define CVs for describing the proton and electron transfer processes. Inspired by the NEB calculation, we select d($O_{\mathrm{sd}}$–$O_{\mathrm{ad}}$) and CN($O_{\mathrm{ad}}$–H). To estimate uncertainties, we train four different machine learning potentials on the same dataset using different initializations for the neural network parameters.^[^ ^21^ ^]^ Our results are then obtained as an average over four independent OPES simulations with these potentials (see Figures S6 and S7).

**Figure 2:**
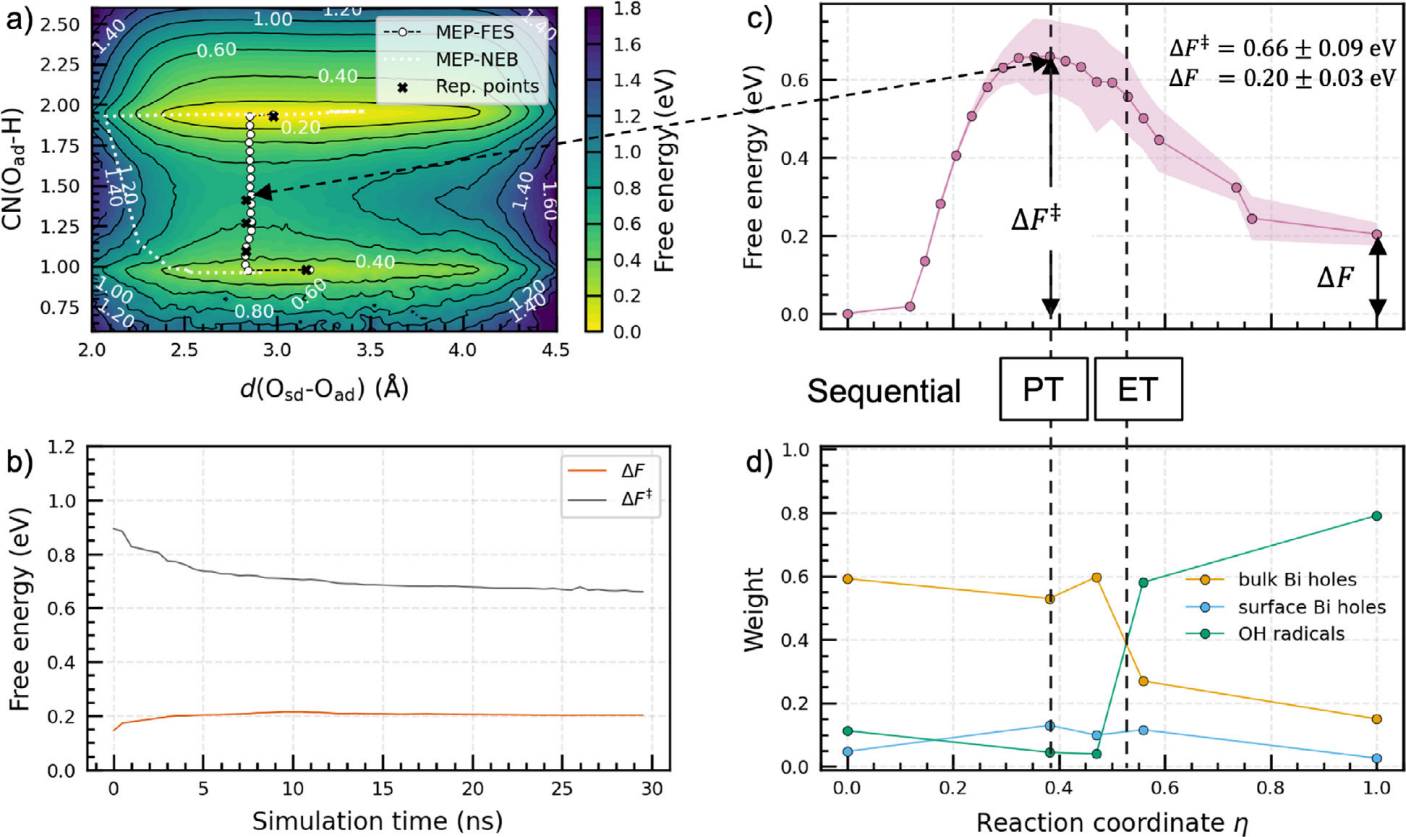
a) Free‐energy surface averaged over four independent simulations involving different machine learning potentials. The minimum energy path on the free‐energy surface (MEP‐FES) is indicated by white circles. The minimum energy path obtained from the nudged‐elastic‐band calculation (MEP‐NEB) is also given through a white dotted curve. b) Evolution of the reaction free energy and the free‐energy barrier as a function of simulation time. c) Free‐energy profile along the MEP‐FES, where $\Delta F^{‡}$ indicates the barrier and ΔF the reaction energy. The shaded area indicates the standard deviation resulting from the four independent simulations. d) Weights for three types of holes, evaluated with one of the MLPs at the representative points (black crosses) in panel (a). In (c) and (d), we use a normalized reaction coordinate η along the path shown in (a).

The free‐energy surface spanned by the two selected CVs in Figure 2a is constructed through the use of 600,000 configurations in each OPES simulation. We find the minimum energy path on the averaged free‐energy surface (MEP‐FES) using the string method.^[^ ^39^ ^]^ The free‐energy profile along the MEP‐FES is shown in Figure 2c. Our results give a free‐energy barrier $\Delta F^{‡}=0.66\pm 0.09$ eV and a reaction free energy ΔF=0.20±0.03 eV. As shown in Figure 2b, it is necessary to continue the simulation for 15 ns to find converged values for both $\Delta F^{‡}$ and ΔF. Remarkably, this result could not be achieved on the time scale of tens of picoseconds generally accessible to *ab initio* molecular dynamics, showing the critical advantage of using machine learning potentials.

To compare with the NEB calculation, the minimum energy path corresponding to the NEB images (MEP‐NEB) is also projected on the same CV space in Figure 2a. We observe that the free‐energy sampling lowers the transition‐state energy on the NEB path by 0.5 eV (see Supporting Information for further discussion). In both paths, the coordination number CN($O_{\mathrm{ad}}$‐H) decreases from 2 to 1. However, a more significant difference is observed for d($O_{\mathrm{sd}}$–$O_{\mathrm{ad}}$). Whereas, in the MEP‐NEB the transition state occurs at $d(O_{\mathrm{sd}}-O_{\mathrm{ad}})≅2.1$ Å, the same O–O distance at the transition state in the MEP‐FES is found at the much larger value of 2.9 Å and remains almost unchanged during the transfer process. From the MEP‐FES, we infer that the CV d($O_{\mathrm{sd}}$–$O_{\mathrm{ad}}$) is irrelevant to the first OER step, indicating that a different reaction mechanism is at play.

### Order of Proton and Hole Transfer

These observations prompt us to investigate the mechanism of the first OER step in greater depth. Generally, the first OER step can proceed via two possible mechanisms, either concerted or sequential proton and electron transfer.^[^ ^40^ ^]^ To reveal the underlying mechanism, it is necessary to locate the proton and electron transfer on the free‐energy surface. The coordination number CN($O_{\mathrm{ad}}$–H) can be used to easily track the proton transfer. In Figure 2a, the free‐energy barrier is observed in correspondence of $\mathrm{CN}(O_{\mathrm{ad}}-H)=1.47$, indicating that the coordinated proton is midway through the dissociation reaction. In contrast, locating the electron transfer is more challenging since it requires electronic‐structure information, which is not directly provided by the machine learning potentials. To retrieve this information, we need to identify representative structural configurations along the free‐energy path. For this purpose, we take five points on the MEP‐FES, marked as black crosses in Figure 2a. These points correspond to CV values of the initial, final, and three intermediate states near the transition point. Next, we need to identify among all the structural configurations those that carry an important weight around these specific CV values.

To identify the relevant structural configurations, we use the following expression to determine their weight ω in contributing to a specific choice of CV values $s_{k}$:

(1) $$\omega \left[s\left(R\right),s_{k}\right]=\frac{G\left[s\left(R\right)-s_{k},\sigma_{\left(s\right)}\right]e^{\beta V(s)}}{{\langle G\left[s\left(R\right)-s_{k},\sigma_{\left(s\right)}\right]e^{\beta V(s)}\rangle }_{V}}$$

where $\omega [s\left(R\right),s_{k}]$ is the weight by which a structural configuration R contributes to a given point $s_{k}$ in CV space. Here, s indicates the CVs of structure R, $G[s\left(R\right)-s_{k},\sigma_{\left(s\right)}]$ a two‐dimensional Gaussian function centered at $s_{k}$ with a bandwidth of $\sigma_{\left(s\right)}$, V(s) the bias deposited during the OPES simulations, ${\langle \cdot \rangle }_{V}$ the average over the biased ensemble, and β the inverse temperature ${\left(k_{B}T\right)}^{-1}$. In the present context, $s_{k}$ is one of the five considered points in Figure 2a. The CVs s correspond to a two dimensional vector [d($O_{\mathrm{sd}}$‐$O_{\mathrm{ad}}$), CN($O_{\mathrm{ad}}$‐H)] and the bandwidths $\sigma_{\left(s\right)}=\left[\sigma_{d},\sigma_{\mathrm{CN}}\right]$. The bandwidths $\sigma_{d}$ and $\sigma_{\mathrm{CN}}$ are taken from the OPES simulations based on the kernel density estimation method.^[^ ^36^ ^]^ For each selected point in CV space, we only retain in our analysis structural configurations carrying a weight larger than 0.003. This results in subsets containing 26 to 108 structural configurations, depending on the considered point in CV space (cf. Table S1).

**Figure 3:**
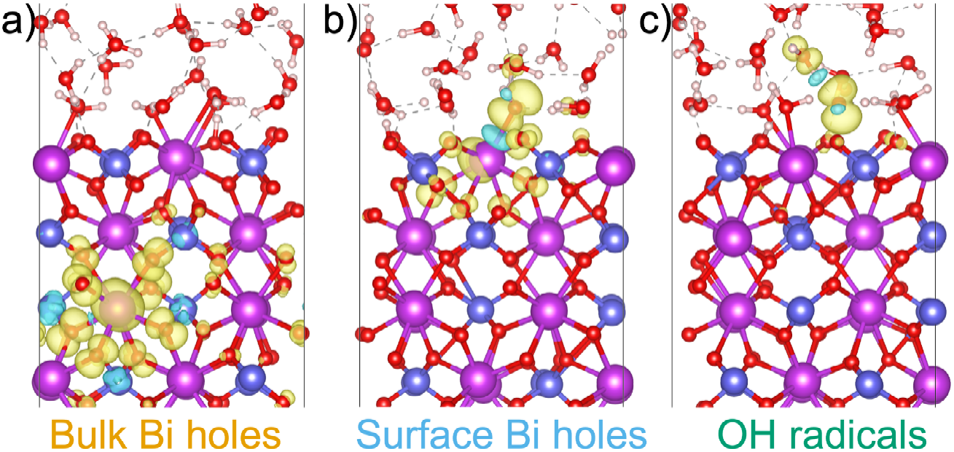
Three distinct types of holes found in the structures sampled by the OPES: holes a) in the bulk, b) at the surface, and c) at a surface OH group (i.e., forming an OH radical). The hole density shown corresponds to the difference between the spin densities in the up and down channels, where the yellow (blue) isosurface indicates positive (negative) spin densities.

For the retained structural configurations, we carry out single‐point hybrid‐functional calculations and analyze their spin densities. Upon visual inspection, we identify three distinct types of holes, as shown in Figure 3. Holes occur either in the bulk (Figure 3a), at the surface (Figure 3b), or at a surface OH group (Figure 3c) originating from an adsorbed water molecule and forming an OH radical. We then plot in Figure 2d, the corresponding weights along the reaction path. We remark that at a given reaction coordinate $s_{k}$ the sum of the weights does not equal 1, because only a subset of the structural configurations is used in the analysis. To locate the electron transfer on the reaction path, we define in Figure 2d, the transition state of the electron transfer as the intersection point between the lines representing bulk holes and OH radicals. This intersection point is found at the value of ∼0.52 for the normalized reaction coordinate η defined in Figure 2d. Alternatively, the transition state can be defined as the intersection point between the lines representing surface holes and OH radicals, leading to η∼0.47. The transition states derived from both definitions suggest that the electron transfer process takes place after the proton transfer process, which is found at η∼0.4 in Figure 2c,d. Interestingly, we observe an increase in the weight of surface Bi holes in the range 0.4<η<0.6. We thus infer that holes are transferred through the mediation of the Bi─$O_{\mathrm{ad}}$ bond (Figure 3b). The role of the Bi atom can further be highlighted by focusing on the Bi─$O_{\mathrm{ad}}$ bond length, which continuously decreases during proton transfer reaching a minimum in correspondence of electron transfer (see Figure S8). The minimum is consistent with either value of η determined above for the electron transfer (η∼0.47 or η∼0.52). This clearly indicates that the Bi─$O_{\mathrm{ad}}$ bond is involved in the electron transfer.

### Mechanism of Proton Transfer

Next, we focus on the mechanism of proton transfer (PT), which according to our calculations corresponds to the bottleneck of the first OER step (cf. Figure 2b). In our simulations, all the adsorbed water molecules at the ${\mathrm{BiVO}}_{4}$‐water interface are intact prior to proton transfer. Through an analysis of the identified structures in correspondence of the final point in Figure 2a, we find that the proton released from the considered water molecule attaches to surface oxygen atoms that are not connected to the same Bi atom ($O_{\mathrm{si}}$) (Figure 1b,c). We do not find any proton released to solvent water molecules, despite the fact that the $pK_{a}$ of a protonated surface oxygen and that of a hydronium ion in the solvent were found to be approximately equal.^[^ ^37^ ^]^

**Figure 4:**
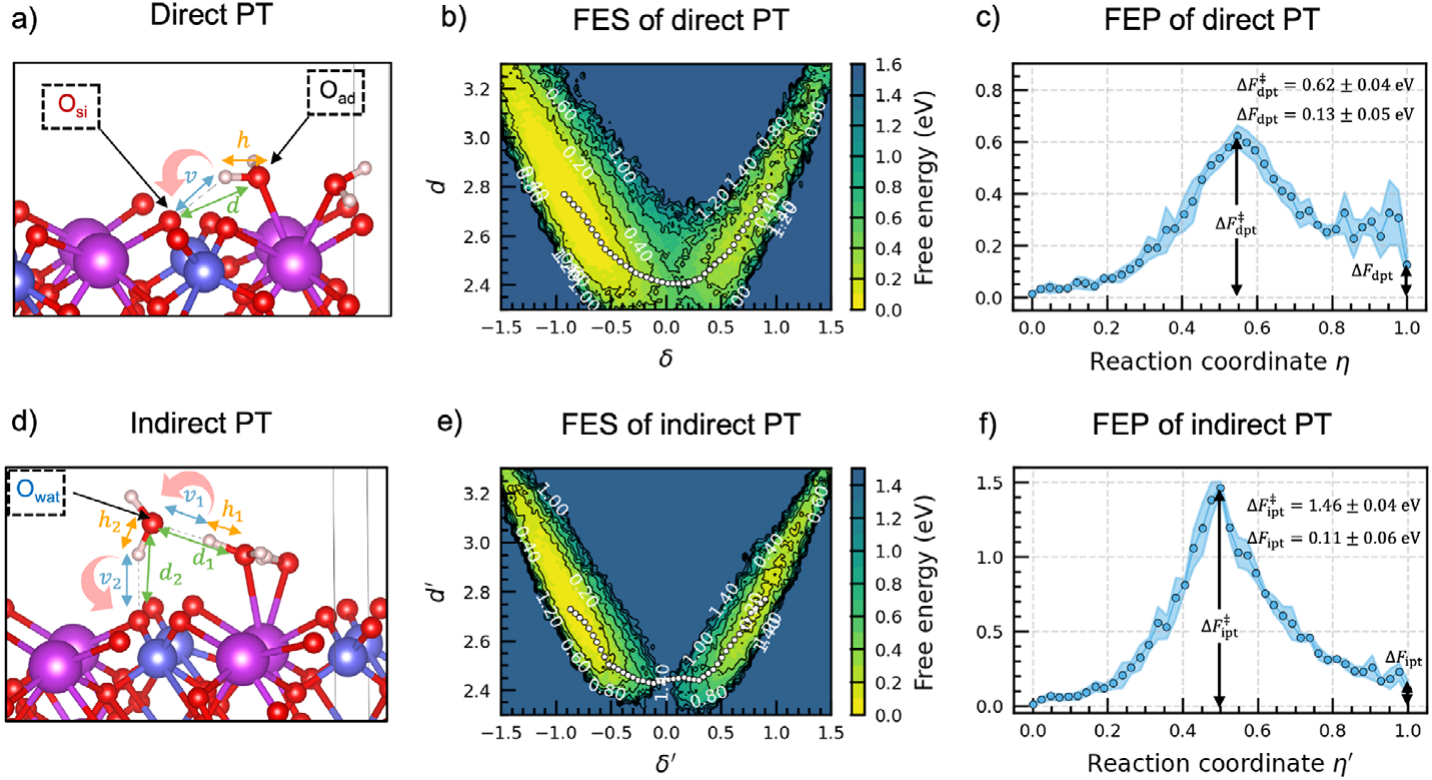
a,d) Interatomic distances used to define the collective variables (CVs) for direct and indirect proton transfer (PT). Irrelevant water molecules are omitted for clarity. For direct PT, the CVs are δ=h−v and $d=d(O_{\mathrm{ad}},O_{\mathrm{si}})$. For indirect PT, the CVs are $\delta^{\prime }=\frac{1}{2}\left[\left(h_{1}-v_{1}\right)+\left(h_{2}-v_{2}\right)\right]$ and $d^{\prime }=\frac{1}{2}\left(d_{1}+d_{2}\right)=\frac{1}{2}\left[d\left(O_{\mathrm{ad}},O_{\mathrm{wat}}\right)+d\left(O_{\mathrm{wat}},O_{\mathrm{si}}\right)\right]$. Red arrows indicate the direction of proton transfer. b, e) Free‐energy surfaces (FES) for direct and indirect PT. The minimum energy paths on the free‐energy surfaces are indicated by white circles. c, f) Free‐energy profiles (FEP) along the minimum energy paths, where $\Delta F_{\mathrm{dpt}}^{‡}\left(\mathrm{ipt}\right)$ and $\Delta F_{\mathrm{dpt}}\left(\mathrm{ipt}\right)$ indicate the barrier and reaction energy of the direct (indirect) PT, respectively. The results in panels (b, c, e, f) are averaged over four independent simulations involving different machine learning potentials. The shaded areas indicate the standard deviation resulting from the four independent simulations.

Two types of proton transfer mechanisms have been proposed in the literature,^[^ ^20^, ^21^, ^23^, ^41^ ^]^ that is, either a direct PT mechanism or an indirect PT mechanism through the mediation of one solvent water molecule (Figure 4a,d). To identify the dominating mechanism, we obtain the corresponding free‐energy surfaces. For this purpose, we define two generalized CVs following previous work,^[^ ^20^, ^21^ ^]^ namely, the distance d between donor and acceptor oxygen atoms and the difference δ between the donor‐hydrogen and acceptor‐hydrogen distances. Specifically, we use δ=h−v and $d=d(O_{\mathrm{ad}}$, $O_{\mathrm{si}})$ for the direct PT (see Figure 4a), while for the indirect PT we take the averages over the two involved PTs, i.e. $\delta^{\prime }=\frac{1}{2}\left[\left(h_{1}-v_{1}\right)+\left(h_{2}-v_{2}\right)\right]$ and $d^{\prime }=\frac{1}{2}\left(d_{1}+d_{2}\right)$ (see Figure 4d). For each structural configuration of the OPES simulations described above, we define hydrogen bonds according to the structural criteria introduced by Luzar and Chandler:^[^ ^42^ ^]^ the distance between the involved O atoms is taken smaller than 3.5 Å and the O–O–H angle smaller than $30^{\circ }$. Configurations are then distinguished depending on whether a direct (Figure 4a) or an indirect (Figure 4d) connection occurs between the adsorbed water molecule and one of the neighboring $O_{\mathrm{si}}$ surface atoms. When neither bonding pattern occurs, we exclude the configuration from our analysis. We construct the free‐energy surfaces by reweighting the CV values through the use of the kernel density estimation method with the corresponding Boltzmann factor ($e^{\beta V}$).^[^ ^36^ ^]^ The bandwidths for the kernel density estimation method are set to 0.003 Å for both d ($d^{\prime }$) and δ ($\delta^{\prime }$), ensuring that the configurations describing the initial, the transition, and the final states are consistent with those in the OPES simulations. The resulting free‐energy surfaces are presented in Figure 4b,e.

To determine which mechanism is likely to proceed, we compare their reaction barriers. We first find the minimum energy paths in the free‐energy surfaces (Figure 4b,e) using the string method.^[^ ^39^ ^]^ Then, we plot the free‐energy profiles along the minimum energy paths for the direct (Figure 4c) and the indirect PT (Figure 4f). For the barrier of the direct ($\Delta F_{\mathrm{dpt}}^{‡}$) and the indirect PT ($\Delta F_{\mathrm{ipt}}^{‡}$), we find 0.62±0.04 and 1.46±0.04 eV, respectively. The ratio of the reaction rates of the two types of PT mechanisms is $r_{\mathrm{ipt}}/r_{\mathrm{dpt}}=\exp\left[-\beta \left(\Delta F_{\mathrm{ipt}}^{‡}-\Delta F_{\mathrm{dpt}}^{‡}\right)\right]=6.2\times 10^{-15}$. This result indicates that the direct PT mechanism dominates the proton transfer process. We remark that the reweighted free‐energy barrier for direct PT is consistent with the overall barrier found in the OPES simulations (Figure 2), further confirming that the PT reaction is the rate‐limiting mechanism.

To characterize the dynamics of the hydrogen‐bond network, we analyze the number of hydrogen bonds accepted by the donor $H_{2}O_{\mathrm{ad}}$ and the acceptor $O_{\text{si}}$ during proton transfer. Our analysis reveals that the number of accepted hydrogen‐bonds on $O_{\text{si}}$ increases at the transition state of both direct and indirect proton transfer processes. This result indicates that a denser hydrogen‐bond network at $O_{\text{si}}$ facilitates proton mobility (see Supporting Information for details).

## Discussion

**Figure 5:**
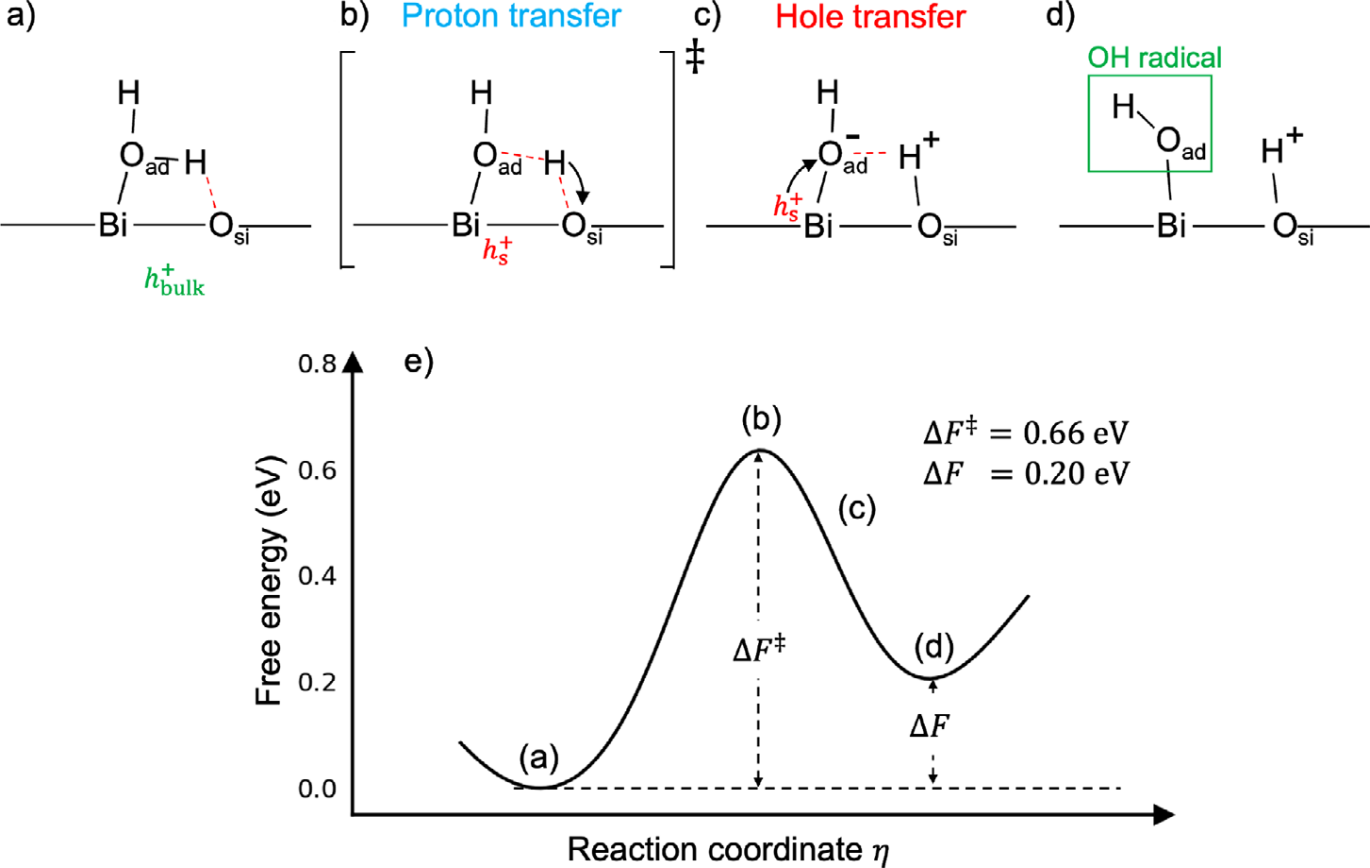
Mechanism of the first proton‐coupled electron transfer (PCET) step. In the first PCET step, key states are a) the initial state, b) the transition state corresponding to the proton transfer, c) an intermediate state for the electron transfer, and d) the final state. $h_{\mathrm{bulk}}^{+}$ indicates the hole in the bulk, $h_{s}^{+}$ the hole at the surface, $O_{\mathrm{ad}}$ the oxygen atom of the adsorbed water molecule, $O_{\mathrm{si}}$ the surface oxygen atom that is not connected to the same Bi atom. e) Schematic free‐energy profile where the values of $\Delta F^{‡}$ and ΔF are taken from Figure 2. The hole transfer is barrierless.

The full mechanism of the first PCET step is summarized in Figure 5. In the initial state, a photo‐generated hole $h_{\mathrm{bulk}}^{+}$ scatters in bulk ${\mathrm{BiVO}}_{4}$ (Figure S9) and an adsorbed water molecule forms a hydrogen bond with a nearby surface oxygen atom $O_{\mathrm{si}}$ (Figure 5a). Next, the proton of the adsorbed water molecule hops to the $O_{\mathrm{si}}$ atom. At the same time, the bulk hole ($h_{\mathrm{bulk}}^{+}$) localizes on the surface Bi atom, becoming a surface hole ($h_{s}^{+}$) (Figure 5b). The proton transfer terminates with $O_{\mathrm{ad}}H^{-}$ and $O_{\mathrm{si}}$–H (Figure 5c). Finally, the surface hole ($h_{s}^{+}$) transfers to the $O_{\mathrm{ad}}H^{-}$ forming an $O_{\mathrm{ad}}H^{.}$ radical (Figure 5d). The schematic free‐energy profile including these states is outlined in Figure 5e. Compared to the overall free‐energy profile (Figure 2c), Figure 5e associates the key states occurring along the reaction path with the configurations shown in Figure 5a–d. In particular, the barrier in Figure 5e is determined by proton transfer, whereas electron transfer appears barrierless. This is consistent with kinetic‐isotope experiments^[^ ^34^, ^35^ ^]^ and suggests that favoring proton transfer could improve the OER performance. Furthermore, the identification of this mechanism allows us to understand the photoelectrochemical performance of other Bi‐based oxides. A very recent computational work^[^ ^43^ ^]^ showed that surface Bi‐rich ${\mathrm{BiVO}}_{4}$ greatly favors the dissociation of adsorbed water molecules (i.e., the proton transfer reaction) compared to surface stoichiometric ${\mathrm{BiVO}}_{4}$. The mechanism identified here then naturally explains the observation of boosted photocurrent reported at surface Bi‐rich ${\mathrm{BiVO}}_{4}$.^[^ ^44^ ^]^

It is of interest to compare the first PCET step at the ${\mathrm{BiVO}}_{4}$ interface with that at the anatase ${\mathrm{TiO}}_{2}$ interface (see also the Supporting Information).^[^ ^17^ ^]^ Both materials share the common feature that the proton transfer precedes the electron transfer and determines the rate‐limiting step. However, the proton‐transfer barrier for ${\mathrm{BiVO}}_{4}$ estimated in this work (0.66±0.09 eV) is found to be higher than for anatase (0.1 eV to 0.5 eV), as calculated in previous works.^[^ ^17^, ^45^ ^]^ For rutile^[^ ^13^ ^]^ and anatase,^[^ ^19^ ^]^ the first PCET step has been found to be the rate‐limiting step of the full OER process. Under the assumption that this also holds for ${\mathrm{BiVO}}_{4}$, the higher reaction barrier rationalizes its lower turnover frequency compared to anatase at the same surface hole density.^[^ ^5^ ^]^ In addition, alkaline conditions would facilitate the proton transfer reaction and hence the OER at the ${\mathrm{BiVO}}_{4}$ interface. Indeed, transition‐metal oxides, such as ${\mathrm{Fe}}_{2}O_{3}$, ${\mathrm{TiO}}_{2}$, and ${\mathrm{MnO}}_{2}$, increase their OER turnover frequencies when the pH of the electrolytes lies beyond the point of zero charge.^[^ ^5^, ^46^, ^47^ ^]^

Despite ${\mathrm{BiVO}}_{4}$ and anatase showing the same sequential order between proton and electron transfers, the underlying atomistic mechanisms of these processes are different. For the proton transfer, the direct mechanism dominates the first PCET step at the ${\mathrm{BiVO}}_{4}$–water interfaces, whereas the indirect mechanism has been observed for the anatase ${\mathrm{TiO}}_{2}$(101)–water interface.^[^ ^21^ ^]^ Regarding the electron transfer, the hole at the ${\mathrm{BiVO}}_{4}$–water interface transfers through the mediation of the Bi─$O_{\mathrm{ad}}$ bond (Figure 3b). At variance, at the anatase–water interface the hole transfer proceeds directly from a surface oxygen atom to the oxygen atom of a nearby adsorbed water molecule.^[^ ^19^ ^]^ The different electron transfer processes can be understood by analyzing the electronic structures of the valence bands. In ${\mathrm{TiO}}_{2}$, the valence band and the hole polaron mostly carry O 2p character.^[^ ^48^ ^]^ In contrast, the valence band^[^ ^49^ ^]^ of ${\mathrm{BiVO}}_{4}$ consists of Bi 6s and O 2p orbitals, making the hole transfer via the Bi─$O_{\mathrm{ad}}$ bond possible. Similarly, at ${\mathrm{Fe}}_{2}O_{3}$–water interface, a hole transfer occurring through the surface transition‐metal atom has been reported in both computational^[^ ^12^ ^]^ and experimental^[^ ^50^ ^]^ studies.

It is in order to discuss whether the mechanism identified in our simulations under the condition of 1 hole ${\mathrm{nm}}^{-2}$ also applies to standard 1 sun irradiation conditions, corresponding to 5 holes ${\mathrm{nm}}^{-2}$. For ${\mathrm{Fe}}_{2}O_{3}$, transient absorption experiments^[^ ^32^ ^]^ revealed that the OER exhibits a third‐order behavior in the surface hole density. This dependence arises from concentration effects associated with surface holes, but the underlying mechanism has been shown to remain the same throughout the range of hole densities exhibiting third‐order behavior.^[^ ^12^ ^]^ Similarly, for ${\mathrm{BiVO}}_{4}$, a third‐order dependence is observed for surface hole densities ranging between 1 and 5 holes ${\mathrm{nm}}^{-2}$.^[^ ^32^ ^]^ This suggests that the mechanism identified in this work remains valid up to 1 sun irradiation conditions.

## Conclusion

In summary, we combine on‐the‐fly probability enhanced‐sampling (OPES) and machine learning potentials to study the free‐energy surface of the first OER step at ${\mathrm{BiVO}}_{4}$–water interfaces. Through the minimum energy path on the free‐energy surface, our simulation reveals the detailed proton and electron transfer mechanisms associated with the water oxidation process. Proton transfer precedes electron transfer and determines the barrier of the reaction, while electron transfer occurs without any barrier. We estimate the reaction barrier at 0.66±0.09 eV and the reaction energy at 0.20±0.03 eV. By analyzing and reweighting the sampled configurations, we identify a mechanism in which the proton transfers to a surface O atom upon the formation of a hydrogen bond without the involvement of solvent water molecules. Next, the hole density hops to the adsorbed hydroxide via a surface Bi atom. Our work demonstrates that the present methodological framework can be instrumental to unravel the mechanisms of proton‐coupled electron transfer reactions at aqueous transition‐metal‐oxide interfaces.

## Conflict of Interests

The authors declare no conflict of interest.

## Supporting information

Supporting Information

## Data Availability

The data that support the findings of this study are available in Materials Cloud (https://archive.materialscloud.org/record/2025.75) with doi: 10.24435/materialscloud:3k‐9k
