# Supplementary material for: Mechanism of First Proton‐Coupled Electron Transfer of Water Oxidation at the BiVO4–Water Interface
Source: Angew Chem Int Ed Engl. 2025 May 24;64(32):e202507071. doi: 10.1002/anie.202507071 (PMC12322655; doi:10.1002/anie.202507071)
Supplement: Supplementary file 1 — Supporting Information [file ANIE-64-e202507071-s001.pdf]

Supporting Information: Mechanism of First Proton-Coupled Electron Transfer of Water Oxidation at the $BiVO_4$-Water Interface

Yong-Bin Zhuang* and Alfredo Pasquarello

Chaire de Simulation à l'Echelle Atomique (CSEA), Ecole Polytechnique Fédérale de Lausanne (EPFL), CH-1015 Lausanne, Switzerland

E-mail: yongbin.zhuang@epfl.ch

Contents

S1 Computational Methods S3

Adopted hybrid functional S3

Machine learning potentials S5

Free-energy sampling scheme S6

S2 Number of adsorbed water molecules at surface Bi atoms S9

S3 Water density profile S11

S4 Energy profile with static water S12

S5 Evolution of collective variables in the NEB calculation S15

S6 Errors of the machine learning potentials on the testing sets S16

S7 Free energy surfaces obtained with the four machine learning potentials S17

S8 Bi−O_{ad} bond length during PCET S18
S9 Hole densities in the oxide for selected configurations S19
S10 Evolution of the number of hydrogen bonds S20
S11 Comparison between the mechanisms at $BiVO_4$ and anatase TiO_2 . . . . S22
References S23

S1 Computational Methods

Adopted hybrid functional

Semilocal approximations to density-functional theory, such as the Perdew-Burke-Ernzerhof approximation of the exchange-correlation energy,[1] generally provide a satifactory description of the atomic structure at oxide-water interfaces.[2,3] However, this level of theory suffers from the self-interaction error leading to underestimated band gaps and delocalized excess holes.[4] One way of overcoming these deficiencies is to resort to hybrid functionals, in which a fraction α of semilocal exchange is replaced with Fock exchange. Thus, we here consider PBE0(α) hybrid functionals, which depend on a single mixing parameter.[5,6]

Dielectric-dependent hybrid functionals (DDH) are constructed nonempirically on the basis of the dielectric response. In the simplest DDH scheme, the parameter α of the hybrid functional is determined through the inverse high-frequency dielectric constant, i.e. $\alpha = \epsilon_\infty^{-1}$.[7,8] These material-specific functionals are well established for bulk materials and have been demonstrated to give a good description of the electronic structure.[9–12]

Recently, hybrid functionals with spatially varying dielectric-dependent α parameters have been proposed for predicting electronic-structure properties of heterogeneous interfaces and surfaces.[13,14] However, it is unclear whether these functionals can be used for calculating forces, which are required for evolving the atomic positions in molecular dynamics simulations. Therefore, we here resort to a single α parameter for describing the full interface system.

We determine the parameter α of the hybrid functional to be used at the $BiVO_4$-water interface through the following scheme. First, using PBE0(α) with a starting value of $\alpha = 0.40$ (ref. 15) we calculate the spatially varying ϵ_∞ at this interface through the method of Giustino and Pasquarello[16] applied to one configuration of the $BiVO_4$-water interface extracted from a molecular dynamics simulation.[17] It is sufficient to use a single snapshot, as it has been shown that the dielectric constant depends weakly on configuration, with

an observed variation of only 0.02 along the molecular dynamics trajectory for a model system containing 32 water molecules.[18] This provides us with an atomic-scale dielectric-constant profile, from which we infer the dielectric constants of the water and $BiVO_4$ interface components, namely $\epsilon_\infty^{\text{water}}$ and $\epsilon_\infty^{BiVO_4}$, respectively. We then obtain an updated value of the parameter α from the average value of $\alpha^{\text{water}} = (\epsilon_\infty^{\text{water}})^{-1}$ and $\alpha^{BiVO_4} = (\epsilon_\infty^{BiVO_4})^{-1}$, i.e.,

$$\alpha = \frac{1}{2}(\alpha^{\text{water}} + \alpha^{BiVO_4}), \quad \text{(S1)}$$

following the averaging scheme put forward in ref. 16. Since the calculated dielectric constants $\epsilon_\infty^{\text{water}}$ and $\epsilon_\infty^{BiVO_4}$ depend on the parameter α, we iterate this procedure until self-consistency is reached. Three iterative steps turn out to be sufficient yielding $\alpha = 0.44$. This value is close to $\alpha = 0.47$ for optimally describing various localized states in liquid water[18] and to $\alpha = 0.45$ derived from a comparison with results from many-body perturbation theory.[19]

To account for nonlocal van der Waals interactions, we add the rVV10 functional[20,21] to the hybrid functional with $\alpha = 0.44$. The empirical parameter b of the rVV10 functional is set to 4.9 to correctly reproduce the experimental density and structural properties of liquid water. This value is obtained by linear extrapolation of results from previous studies where the optimal b parameters were found to be 9.3 and 5.3 for $\alpha = 0$ and $\alpha = 0.40$, respectively.[15,22] Hence, all the calculations at the $BiVO_4$-water interface are carried with the functional PBE0($\alpha = 0.44$)+rVV10($b = 4.9$).

The hybrid-functional calculations are carried out with the freely available CP2K code,[23] which combines Gaussian and plane-wave basis sets. The orbitals are represented in a Gaussian-type double-ζ basis with one set of polarization functions (DZVP), while an auxiliary plane-wave basis set with an energy cutoff of 600 Ry is used to expand the electron density.[24,25] The core electrons are described by analytic Goedecker-Teter-Hutter pseudopotentials.[26] The self-consistent-field optimization of the wave functions uses the orbital trans-

formation algorithm,[27] with a convergence threshold set to 1×10^{-5} a.u. As convergence test, we tighten the convergence threshold from 1.0×10^{-5} to 1.0×10^{-6} for one representative configuration. The total energy and the root-mean-squared difference of the atomic forces are found to differ by only 7.0×10^{-3} eV and 2.0×10^{-6} eV/Å, respectively. The adopted threshold is thus considered to be sufficiently stringent. The auxiliary density matrix method (ADMM)[28,29] is used to speed up the calculation of exchange integrals. For this, we employ the following auxiliary basis sets: cFIT3 for O and H atoms, cFIT6 for Bi atoms, and cFIT10 for V atoms.[30]

Nuclear quantum effects (NQEs) have been shown to affect the free-energy barriers of proton transfer reactions.[31] For instance, these effects have been found to affect the calculated free-energy barrier in the case of water dissociation on a Pt surface by ~ 0.05 eV at 300 K.[32] Similarly, NQEs have been estimated to affect the energy barriers by 0.02 eV in the case of excess proton transfer in water.[33] In the scheme applied in this work, residual errors in the calculated free-energy barriers are on the order of 0.1 eV (*vide infra*), justifying the neglect of NQEs in the present simulations.

The hole is introduced in the calculation by removing one electron from the neutral system. The resulting positive charge is compensated by a uniform background in the supercell. We estimated finite-size effects due to this background and to the periodic image charges through the scheme detailed in ref. 34. The calculated free-energy barrier is affected by less than 0.05 eV, whereas the finite-size effects lead to an increase of the reaction free energy by less than 0.1 eV. Given their small size, these corrections have not been included in the presented results.

Machine learning potentials

The training datasets are collected using the open-source ai^2-kit package, which is an extension of the concurrent learning package DP-GEN.[35,36] During the collection process, the "Training", "Exploration", and "Labeling" stages are performed iteratively to ensure the

Table S1: Details and errors of the viable training datasets. N_{atoms} is the number of atoms and N_{conf} is the number of configurations in the datasets. The accuracy of the machine learning potentials is determined by the average root mean square errors of potential energies (E_{RMSE}) and forces (F_{RMSE}). Interface models 1 and 2 have 312 and 360 atoms, respectively.

	L_x (Å)	L_y (Å)	L_z (Å)	N_{atoms}	N_{conf}	E_{RMSE} (meV/atom)	F_{RMSE} (meV/Å)
Interface model 1	10.39	10.18	36.17	312	873	2.40	168.89
Interface model 2	10.39	10.18	36.17	360	1152	2.30	171.27

reliability of the datasets. In the “Training” stage, machine learning potentials (MLPs) are trained using the open-source DeePMD-kit package.[37] In the “Exploration” stage, on-the-fly probability enhanced sampling (OPES) is performed to explore the configuration space of the $BiVO_4$-water interface system through the combined use of the open-source LAMMPS[38] and PLUMED packages.[39] In the “Labeling” stage, hybrid-functional calculations are carried out with the CP2K package. The initial dataset includes 50 structures taken from a previously generated molecular dynamics trajectory.[17,40] In the end, the viable datasets contain 2025 structures in total. The details of the datasets are listed in the Table S2.

Free-energy sampling scheme

Molecular dynamics simulations are performed using the LAMMPS code[38] driven by the MLPs. All the simulations sample the NVT ensemble with a temperature of 350 K to ensure a frank diffusion of water.[15] The temperature is controlled by the Nosé-Hoover thermostat with a coupling constant of 0.1 ps. The timestep is set to 0.5 fs. We use OPES[41,42] to explore the free-energy surface of the first PCET step of the OER. This sampling approach provides access to the kinetics and to the microscopic transition mechanism between different metastable states. OPES converges more rapidly and has fewer bias oscillations than the commonly used metadynamics method.[41] Thus, this scheme is particularly suitable for

accurately estimating a free-energy surface (FES).[41]

We sample the free-energy surface of the OER by predefining two collective variables (CVs). The first CV is the distance between one specific oxygen atom of an adsorbed water molecule (O_{ad}) and one of its neighboring surface oxygen atoms (O_{sd}), see Figure 1 in the main text. The second CV is the coordination number of the selected O_{ad} to hydrogen. In OPES, the CVs must be continuous and differentiable across configurational space. For this reason, we adopt the smooth version of coordination number as described in the PLUMED manual.[39,43] The C_i^{B} calculates the number of atoms of species B within a sphere of radius r_0 centered on atom i according to:

$$C_{i\in \mathrm{A}}^{\mathrm{B}} = \sum_{j\in \mathrm{B}}^{N_j} \frac{1-\left(\frac{d_{ij}}{r_0}\right)^n}{1-\left(\frac{d_{ij}}{r_0}\right)^m} \tag{S2}$$

where the d_{ij} is the distance between the atom i of species A (O_{ad}) and atom j of species B (H). N_j is the number of B atoms under consideration. The m and n control the smoothness of the CV and are set to 24 and 12, respectively. The r_0 is set to 1.3 Å for O−H bonds, approximately corresponding to the first minimum in the oxygen-hydrogen radial distribution function.[15] To prevent water exchange processes of the adsorbed water molecule occurring at the Bi surface atom, we further apply an harmonic wall for Bi-O bond lengths exceeding 3.5 Å. This constraint does not affect the equilibrium motion which occurs at 2.61 ± 0.05 Å. The harmonic wall is solely applied to the water molecule that undergoes water oxidation. Remaining adsorbed water molecules are allowed to proceed water exchange.

To ensure an accuracy of 0.05 eV for the reaction energy and the free-energy barrier, we proceed as follows. We perform consecutive OPES simulations lasting 5 ns. Our results are considered to be converged when the free-energy variation is less than 0.05 eV over a 5-nanosecond interval. Convergence is achieved at 15 ns. Between 10 and 15 ns, the reaction energy and the free-energy barrier are found to vary by 0.006 eV and 0.022 eV, respectively. A convergence check at 30 ns confirms that the free energies are converged within the required

accuracy.

S2 Number of adsorbed water molecules at surface Bi atoms

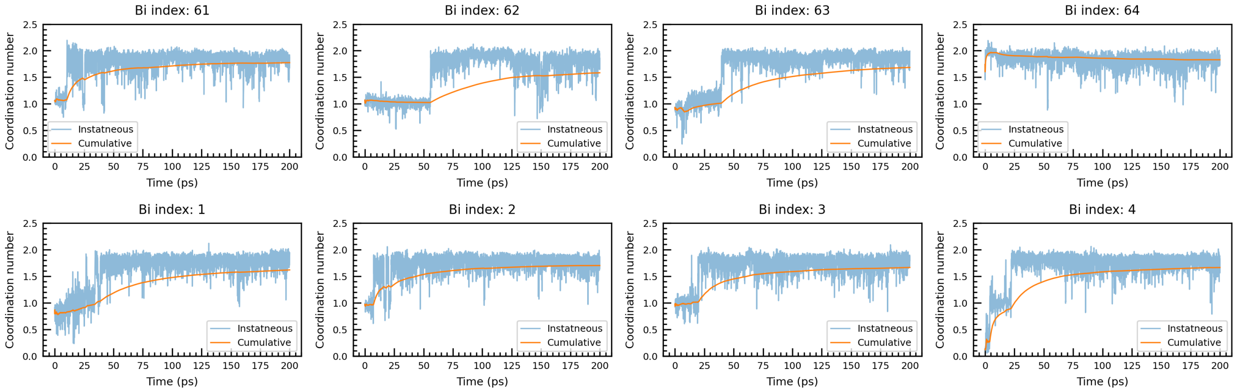

Figure S1: Coordination number of surface Bi atoms to water molecules for the eight surface Bi atoms in the 312-atom interface model containing 56 water molecules.

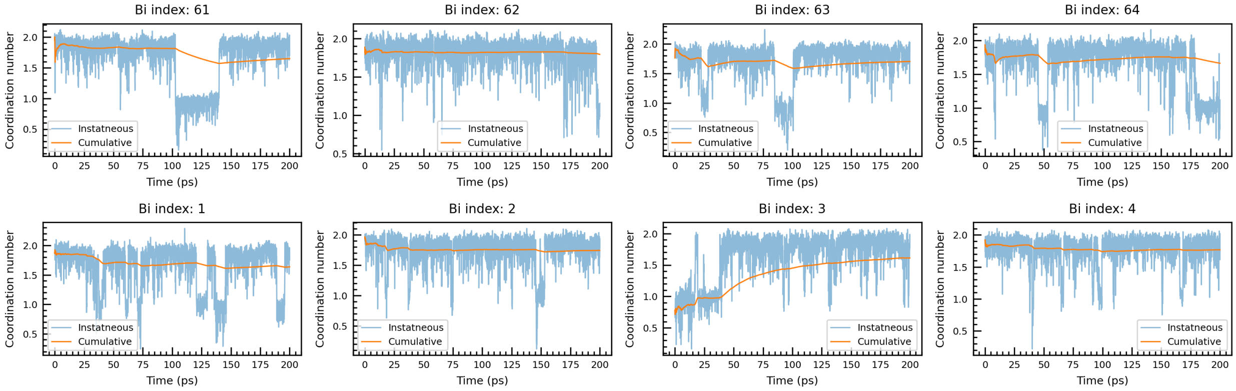

Figure S2: Coordination number of surface Bi atoms to water molecules for the eight surface Bi atoms in the 360-atom interface model containing 72 atoms.

We perform molecular dynamics (MD) simulations without any harmonic restraint on Bi−O bonds to investigate the number of adsorbed water molecules. Initially, we run an unbiased MD simulation with a machine-learning potential for 200 ps for the 312-atom interface model. In Figure S1, we display the coordination number of each surface Bi atom to oxygen atoms of water molecules as a function of simulation time. The coordination number is calculated with the same formula given in eq. 2 of the main text, where the cutoff r_0 is set to

3.2 Å, corresponding to the first minimum in the Bi-O_{ad} radial distribution function reported in ref. 3. In the simulation, we observe the adsorption of one additional water molecule on each surface Bi site. Surface Bi sites with one water molecule correspond to a transient state and are found to survive for timescales ranging from 25 ps to 50 ps. These timescales exceed those of typical *ab initio* MD simulations[2,3,17] and explain why the transient nature of surface Bi atoms bonded to a single water molecule has not been detected.[3,17,44] Additionally, we perform an unbiased MD simulation for a 360-atom interface model in which each surface Bi site is coordinated with two water molecules from the beginning. During the MD simulation, we observe that the number of adsorbed water molecules is generally stable around 2 with only occasional short-term transitions to 1. This phenomenon is attributed to water exchange on the surface Bi atoms.

S3 Water density profile

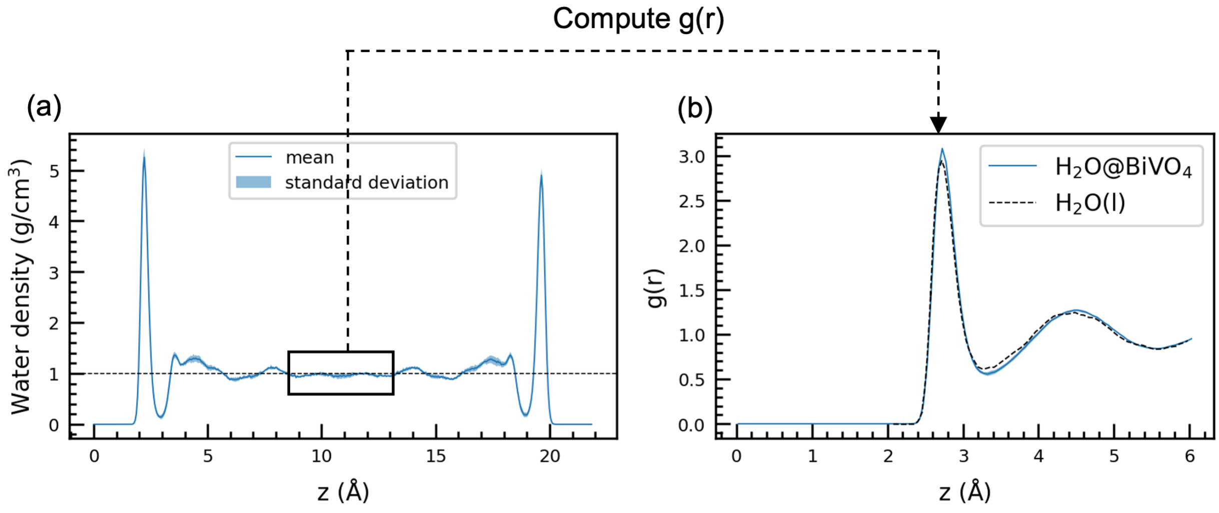

Figure S3: (a) Water density profile obtained from unbiased molecular dynamics simulations with machine learning potentials for the $BiVO_4$(0 1 0)-water interface model containing 360 atoms. The profile corresponds to a time average over a duration of 1 ns and has been obtained as an average over four different machine learning potentials. The standard deviation is calculated from the four independent simulations. (b) Oxygen-oxygen radial distribution functions (RDFs) in the central water layer of 5 Å of the $BiVO_4$-water interface model (H_2O@$BiVO_4$) and of bulk liquid water [H_2O(l)]. The latter RDFs are taken from reference 15.

S4 Energy profile with static water

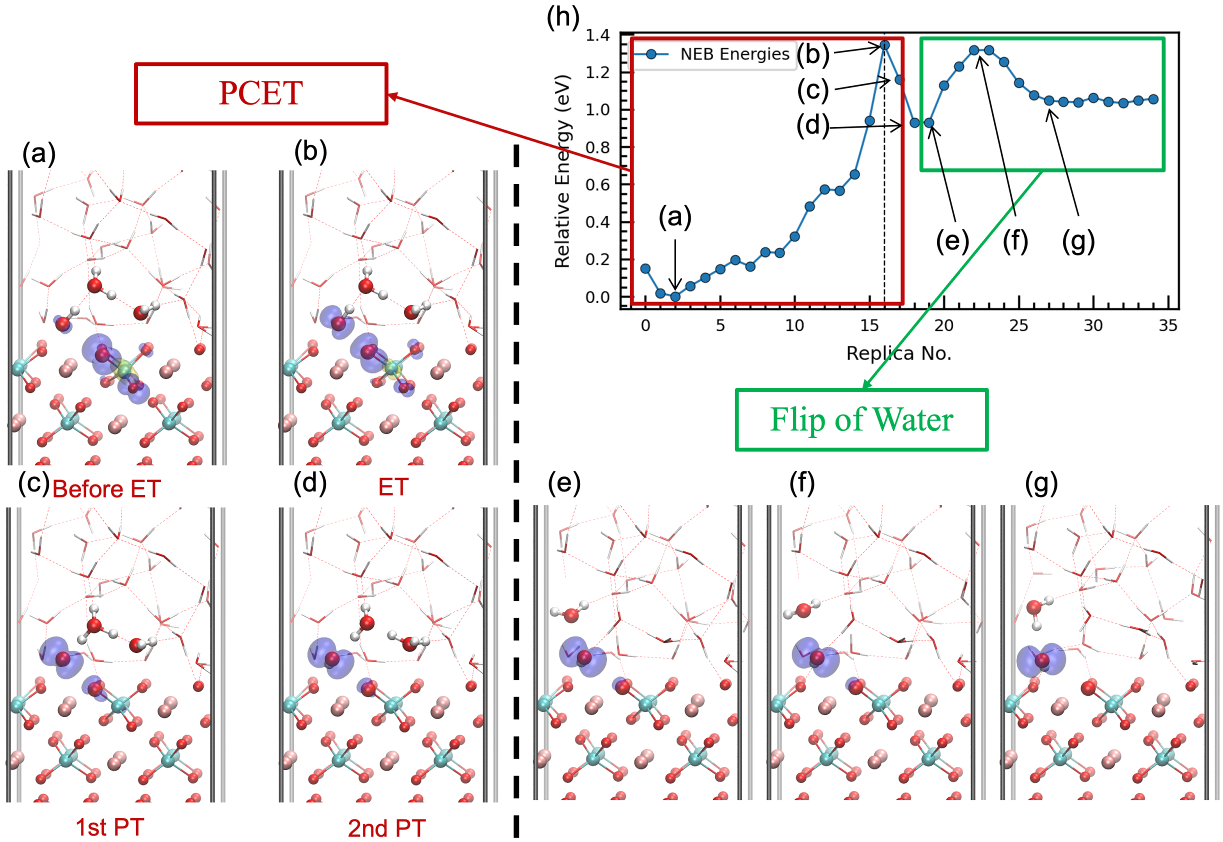

Figure S4: Hole densities as obtained for key images in the NEB calculation. (a-c) Electron and (b-d) proton transfer steps are shown. (e-g) correspond to the subsequent flip of one water molecule in the NEB calculation. (h) Energy profile with respect to the number of NEB images.

We are interested in performing a nudged-elastic band (NEB) calculation in the presence of static water to obtain the energy barrier of the first proton-coupled electron transfer at the $BiVO_4$-water interface. In ref. 45, the NEB calculation was based on a model with one adsorbed water per surface Bi site. Here, we perform a similar NEB calculation for the 360-atom interface model with two adsorbed water molecules per Bi site. In these calculations, we use the PBE0($\alpha = 0.44$) hybrid functional supplemented with nonlocal rVV10($b = 0.49$) density functional to account for van-der-Waals interactions[20,21] (cf. main

text). At the hybrid-functional level, the NEB calculation is computationally demanding. To reduce the cost, we create 35 initial images for the current NEB calculation taking advantage of the NEB trajectory[46] found in ref. 45. We proceed as follows. We first take the three structures corresponding to the initial, the transition, and the final states from the NEB trajectory found previously.[45] After accommodating 16 more water molecules to these images, we optimize their structure. Next, the other images are recreated through linear interpolations. We find the minimum energy path through the climbing-image scheme.[47] The first and last images are fixed during the NEB optimization. In Figure S4, we give the energy profile of the minimum energy path together with the hole densities for key images. Despite the higher number of adsorbed water molecules per Bi site, we find the same mechanism as in ref. 45, i.e. the process is initiated by a hole hopping to an adsorbed water molecule (Figure S4a-b), followed by proton transfer reactions (Figure S4b-d) leading to the formation of a hydronium ion.

The mechanism found with the NEB calculation differs from that revealed by the OPES simulation. The divergence between the two mechanisms could originate from both the initial/final-state configurations and the water dynamics. It is difficult to disentangle the role of these two effects. The final-state configurations in the nudged-elastic band (NEB) calculation and free-energy sampling (FES) are the same, i.e. configurations with a hole remaining on a chemisorbed water molecule. At variance, the initial-state configuration in the NEB calculation contains a hole localized on a surface oxygen atom (Figure S4a), whereas at finite temperature the hole in the initial-state configuration of the FES fluctuates between various positions, being prevalently located at a bulk Bi atom (Figure 3a and Figure S9). The different initial-state configurations could thus be responsible for the observation of different reaction mechanisms. However, the water dynamics can also lead to a sizable variation of the energy surface. Indeed, we can estimate the effect of water dynamics by focusing on the white dotted line on the free-energy surface in Figure 2a. This line corresponds to the projection of the configurations obtained with the NEB calculation onto the two-dimensional

space spanned by the collective variables, CN(O_{ad})-H and d(O_{sd}-O_{ad}). The transition state as found in the NEB calculation is approximately located at (CN=1.9, d=2.1 Å) on the free-energy surface. The free energy at this point amounts to 0.80 eV lower by 0.54 eV compared to the NEB barrier of 1.34 eV. Such a significant change could thus also contribute to the variation of the minimum-energy path.

S5 Evolution of collective variables in the NEB calculation

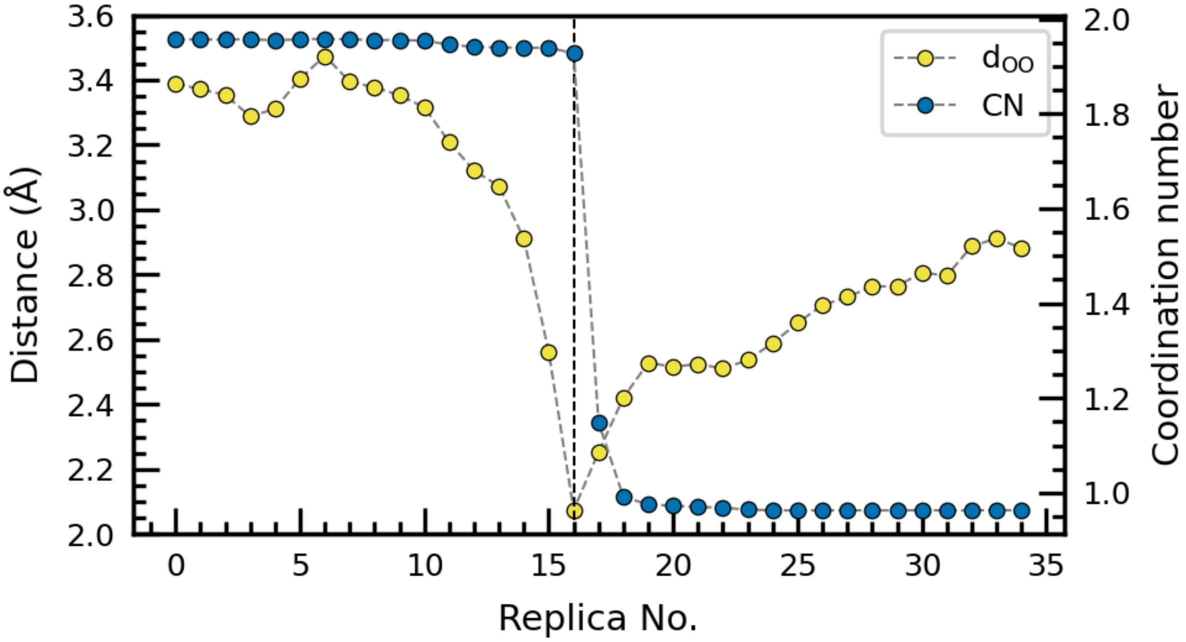

Figure S5: Evolution of the distance d_{OO} between the surface oxygen atom and the oxygen atom in the adsorbed water molecule and of the coordination number CN. The vertical dashed line indicates the transition state found with the NEB calculation.

S6 Errors of the machine learning potentials on the testing sets

Table S2: Root mean square errors (RMSEs) of the energies (E_{RMSE}) and forces (F_{RMSE}) associated with one of the four machine learning potentials (MLPs) with respect to full hybrid functional calculations. The testing datasets considered here correspond to representative structural configurations at specific values of two collective variables (see Figure 2 in the main text). The considered collective variables are the distance between the surface O atom and the O atom of the adsorbed water molecule (d_{OO}) and the number of hydrogen atoms coordinated to the latter O atom (CN). N_{conf} is the number of configurations in the testing datasets. The total weights of the N_{conf} configurations correspond to the sum of weights associated with bulk Bi holes, surface Bi holes, and OH radicals, as given in Figure 2d of the main text. The total weights indicate that subsets corresponding to 70 to 97% of the full set of configurations are being considered. The excluded configurations carry weights lower than 0.003 for the specific collective variables considered (see main text).

d_{OO}	CN	N_{conf}	E_{RMSE} (meV/atom)	F_{RMSE} (meV/Å)	Total weight
2.979	1.930	104	1.69	165.46	0.753
2.831	1.412	101	1.98	172.82	0.705
2.831	1.268	108	2.05	181.74	0.736
2.831	1.095	36	1.47	172.75	0.966
3.155	0.980	26	2.15	164.73	0.968

S7 Free energy surfaces obtained with the four machine learning potentials

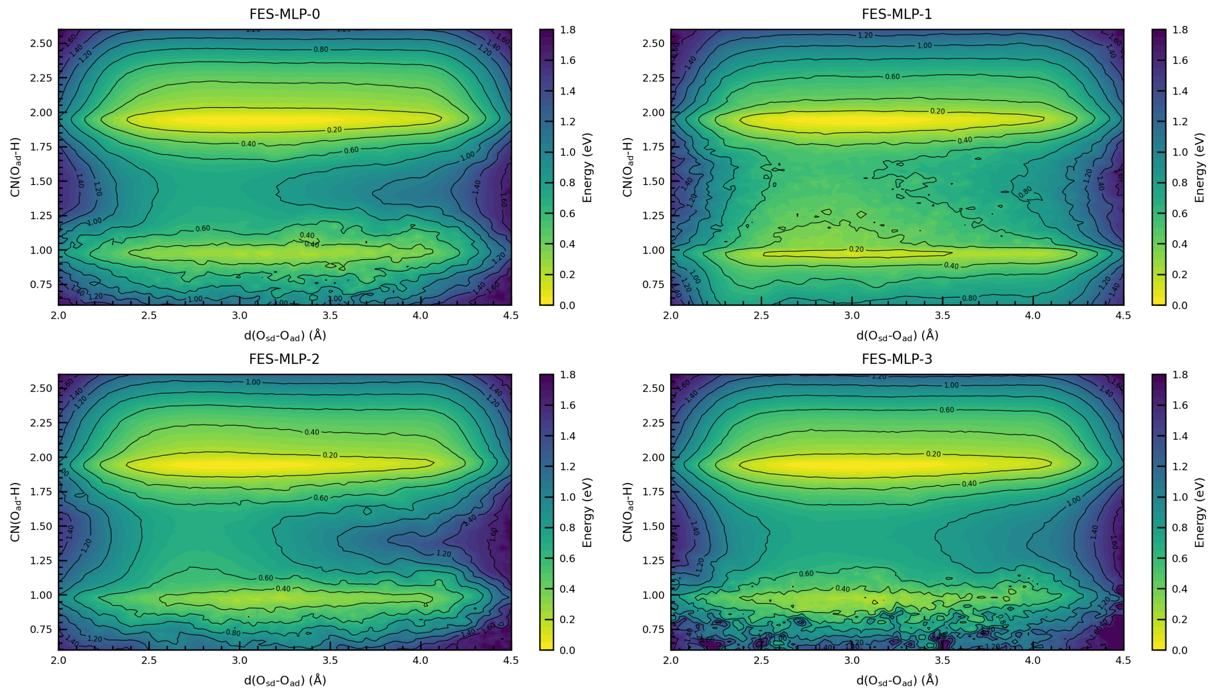

Figure S6: Free energy surfaces obtained from four independent OPES simulations involving four different machine learning potentials.

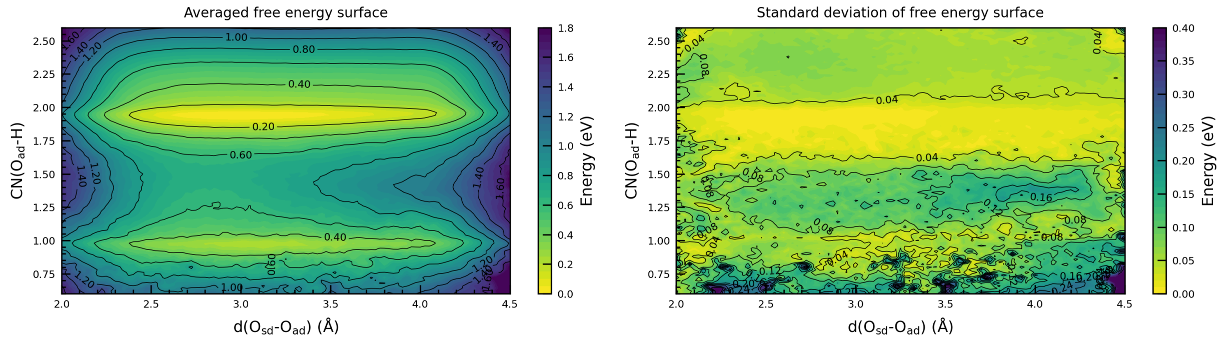

Figure S7: (a) Free energy surface averaged over the four free-energy surfaces given in Figure S6, with (b) corresponding standard deviation.

S8 Bi−O_{ad} bond length during PCET

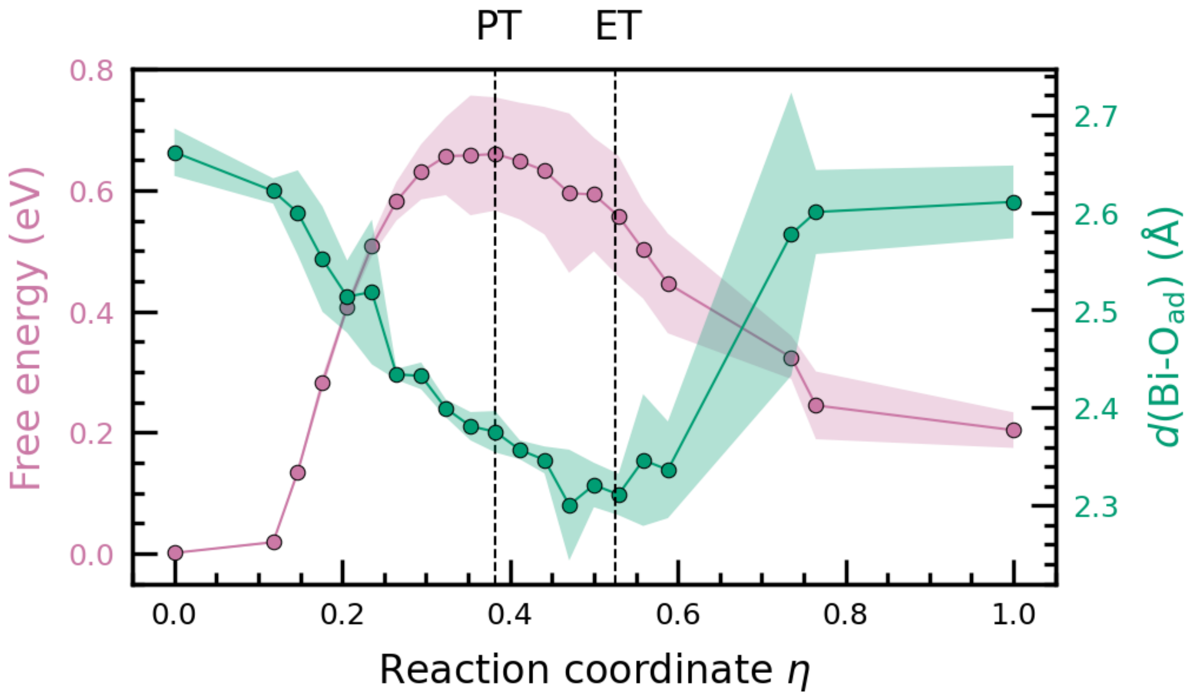

Figure S8: Correlation between the free-energy (left scale) and the Bi−O_{ad} bond length (right scale) along the reaction path identified in Figure 2 of the main text. The vertical lines for PT and ET correspond to those found in Figure 2 of the main text. The shaded areas represent the standard deviations for averages over the four machine learning potentials used.

S9 Hole densities in the oxide for selected configurations

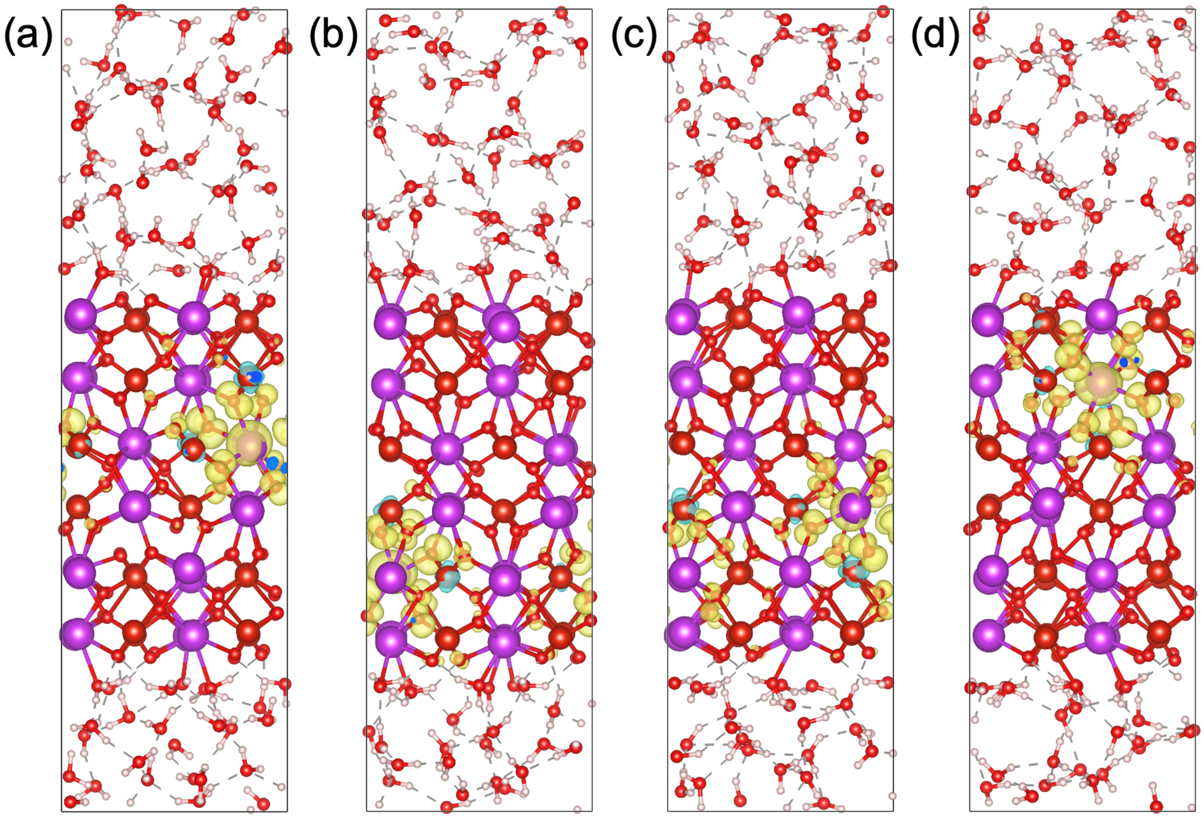

Figure S9: Hole polaron densities for four selected configurations corresponding to the initial state of the first proton-coupled electron transfer step.

We present the hole polaron densities for four selected configurations corresponding to the initial state of the first proton-coupled electron transfer step. The hole densities are located at the different Bi sites, indicating that the holes diffuse easily with thermal fluctuations. This is consistent with the binding energy of 0.11 eV calculated by thermodynamic integration[17,48] and with the low thermal hopping activation energy of 0.09 eV measured in a THz spectroscopy experiment.[49]

S10 Evolution of the number of hydrogen bonds

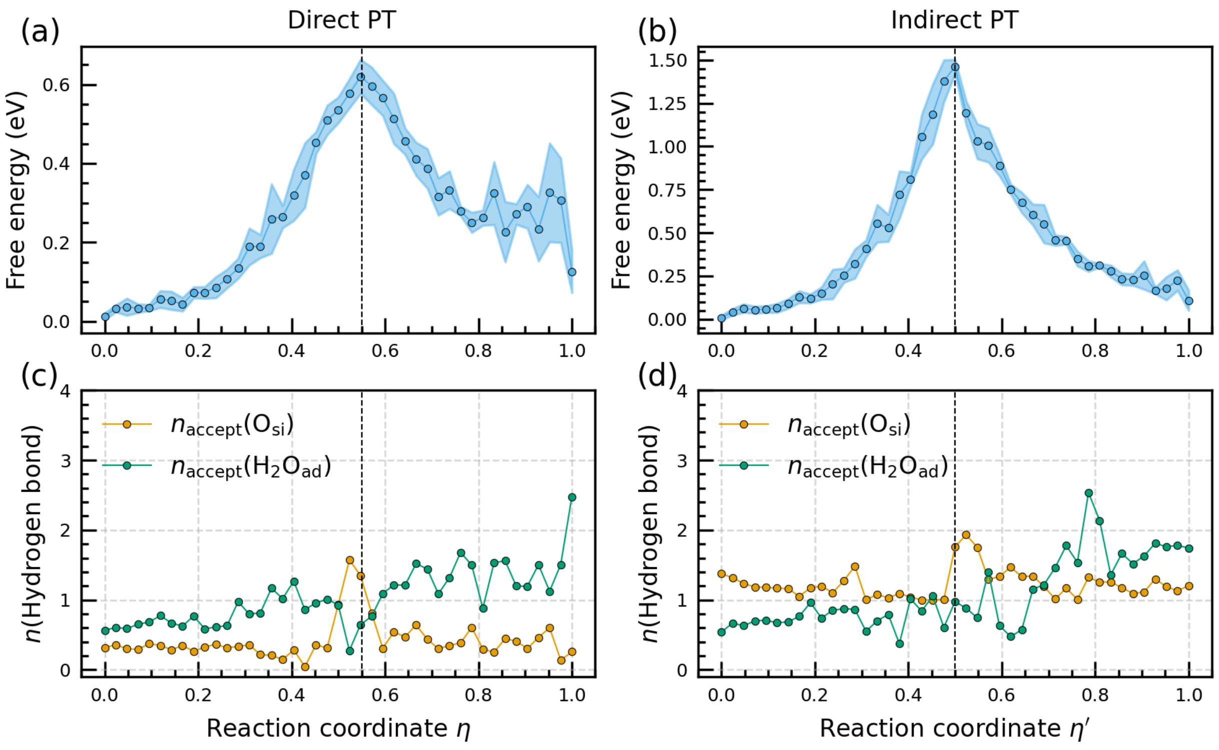

Figure S10: Free-energy profiles for (a) direct and (b) indirect proton transfer. The evolution of number of hydrogen bonds for (c) direct and (d) indirect proton transfer.

To characterize the role of the hydrogen-bond network, we analyze the number of hydrogen bonds accepted by the donor H_2O_{ad} and acceptor O_{si} in the direct and indirect proton transfer reactions. The results are shown in Figure S10.

First, we focus on the evolution of the number of hydrogen bonds accepted by the H_2O_{ad} [$n_{accept}(H_2O_{ad})$]. $n_{accept}(H_2O_{ad})$ in both direct and indirect proton transfer shows similar trends. Prior to proton transfer, $n_{accept}(H_2O_{ad})$ is approximately 0.5, and is found to increases to 1.3-1.7 after proton transfer. In contrast, the number of hydrogen bonds accepted by O_{si} [$n_{accept}(O_{si})$] is different for direct and indirect proton transfer. In the direct proton transfer reaction, $n_{accept}(O_{si})$ starts at 0.3, increases to 1.5 as the reaction reaches the barrier, and then returns to 0.3. In the indirect proton transfer reaction, $n_{accept}(O_{si})$ starts at 1.3, increases to 2.0 as the reaction reaches the barrier, and then returns to 1.3.

The results reveal two interesting features. First, $n_{\mathrm{accept}}(\mathrm{O_{si}})$ increases at the transition state of both direct and indirect proton transfer processes. This result indicates that a denser hydrogen-bond network at $\mathrm{O_{si}}$ facilitates proton mobility. Second, a larger value of $n_{\mathrm{accept}}(\mathrm{O_{si}})$ ($\sim$1.3) is required to activate the indirect proton transfer compared to the direct proton transfer ($\sim$0.3).

S11 Comparison between the mechanisms at $BiVO_4$ and anatase TiO_2

Table S3: Comparison between the mechanisms of the first PCET step at $BiVO_4$ and anatase TiO_2. The results for anatase TiO_2 are taken from reference 50.

Material	$BiVO_4$	anatase TiO_2
Limiting step	proton transfer	proton transfer
Barrier height	0.66 eV	0.2-0.5 eV
Proton transfer type	direct PT	indirect PT
Electron transfer path	from Bi to O	from O to O

References

(1) Perdew, J. P.; Burke, K.; Ernzerhof, M. Generalized Gradient Approximation Made Simple. *Phys. Rev. Lett.* **1996**, *77*, 3865–3868.

(2) Guo, Z.; Ambrosio, F.; Chen, W.; Gono, P.; Pasquarello, A. Alignment of Redox Levels at Semiconductor–Water Interfaces. *Chem. Mater.* **2018**, *30*, 94–111.

(3) Ambrosio, F.; Wiktor, J.; Pasquarello, A. pH-Dependent Surface Chemistry from First Principles: Application to the $BiVO_4$(010)–Water Interface. *ACS Appl. Mater. Interfaces* **2018**, *10*, 10011–10021.

(4) Yang, J.; Falletta, S.; Pasquarello, A. One-Shot Approach for Enforcing Piecewise Linearity on Hybrid Functionals: Application to Band Gap Predictions. *J. Phys. Chem. Lett.* **2022**, *13*.

(5) Perdew, J. P.; Ernzerhof, M.; Burke, K. Rationale for mixing exact exchange with density functional approximations. *J. Chem. Phys.* **1996**, *105*, 9982–9985.

(6) Adamo, C.; Barone, V. Toward reliable density functional methods without adjustable parameters: The PBE0 model. *J. Chem. Phys.* **1999**, *110*, 6158–6170.

(7) Alkauskas, A.; Broqvist, P.; Pasquarello, A. Defect levels through hybrid density functionals: Insights and applications. *Phys. Status Solidi B* **2011**, *248*, 775–789.

(8) Marques, M. A. L.; Vidal, J.; Oliveira, M. J. T.; Reining, L.; Botti, S. Density-based mixing parameter for hybrid functionals. *Phys. Rev. B* **2011**, *83*, 035119.

(9) Skone, J. H.; Govoni, M.; Galli, G. Self-consistent hybrid functional for condensed systems. *Phys. Rev. B* **2014**, *89*, 195112.

(10) Skone, J. H.; Govoni, M.; Galli, G. Nonempirical range-separated hybrid functionals for solids and molecules. *Phys. Rev. B* **2016**, *93*, 235106.

(11) Chen, W.; Miceli, G.; Rignanese, G.-M.; Pasquarello, A. Nonempirical dielectric-dependent hybrid functional with range separation for semiconductors and insulators. *Phys. Rev. Mater.* **2018**, *2*, 073803.

(12) Yang, J.; Falletta, S.; Pasquarello, A. Range-separated hybrid functionals for accurate prediction of band gaps of extended systems. *Npj Comput. Mater.* **2023**, *9*, 108.

(13) Borlido, P.; Marques, M. A. L.; Botti, S. Local Hybrid Density Functional for Interfaces. *J. Chem. Theory Comput.* **2018**, *14*, 939–947.

(14) Zheng, H.; Govoni, M.; Galli, G. Dielectric-dependent hybrid functionals for heterogeneous materials. *Phys. Rev. Mater.* **2019**, *3*, 073803.

(15) Ambrosio, F.; Miceli, G.; Pasquarello, A. Structural, Dynamical, and Electronic Properties of Liquid Water: A Hybrid Functional Study. *J. Phys. Chem. B* **2016**, *120*, 7456–7470.

(16) Giustino, F.; Pasquarello, A. Theory of atomic-scale dielectric permittivity at insulator interfaces. *Phys. Rev. B* **2005**, *71*, 144104.

(17) Wiktor, J.; Pasquarello, A. Electron and Hole Polarons at the $BiVO_4$–Water Interface. *ACS Appl. Mater. Interfaces* **2019**, *11*, 18423–18426.

(18) Bischoff, T.; Reshetnyak, I.; Pasquarello, A. Band gaps of liquid water and hexagonal ice through advanced electronic-structure calculations. *Phys. Rev. Mater.* **2021**, *3*, 023182.

(19) Ambrosio, F.; Guo, Z.; Pasquarello, A. Absolute Energy Levels of Liquid Water. *J. Phys. Chem. Lett.* **2018**, *9*, 3212–3216.

(20) Vydrov, O. A.; Van Voorhis, T. Nonlocal van der Waals density functional: The simpler the better. *J. Chem. Phys.* **2010**, *133*.

(21) Sabatini, R.; Gorni, T.; de Gironcoli, S. Nonlocal van der Waals density functional made simple and efficient. *Phys. Rev. B* **2013**, *87*, 041108.

(22) Miceli, G.; de Gironcoli, S.; Pasquarello, A. Isobaric first-principles molecular dynamics of liquid water with nonlocal van der Waals interactions. *J. Chem. Phys.* **2015**, *142*, 034501.

(23) Kühne, T. D. et al. CP2K: An electronic structure and molecular dynamics software package - Quickstep: Efficient and accurate electronic structure calculations. *J. Chem. Phys.* **2020**, *152*, 194103.

(24) VandeVondele, J.; Hutter, J. Gaussian basis sets for accurate calculations on molecular systems in gas and condensed phases. *J. Chem. Phys.* **2007**, *127*, 114105.

(25) Lippert, G.; Hutter, J.; Parrinello, M. A hybrid Gaussian and plane wave density functional scheme. *Mol. Phys.* **1997**, *92*, 477–487.

(26) Goedecker, S.; Teter, M.; Hutter, J. Separable dual-space Gaussian pseudopotentials. *Phys. Rev. B* **1996**, *54*, 1703–1710.

(27) VandeVondele, J.; Hutter, J. An efficient orbital transformation method for electronic structure calculations. *J. Chem. Phys.* **2003**, *118*, 4365–4369.

(28) Guidon, M.; Schiffmann, F.; Hutter, J.; VandeVondele, J. Ab initio molecular dynamics using hybrid density functionals. *J. Chem. Phys.* **2008**, *128*, 214104.

(29) Guidon, M.; Hutter, J.; VandeVondele, J. Robust Periodic Hartree-Fock Exchange for Large-Scale Simulations Using Gaussian Basis Sets. *J. Chem. Theory Comput.* **2009**, *5*, 3010–3021.

(30) Guidon, M.; Hutter, J.; VandeVondele, J. Auxiliary Density Matrix Methods for Hartree-Fock Exchange Calculations. *J. Chem. Theory Comput.* **2010**, *6*, 2348–2364.

(31) Markland, T. E.; Ceriotti, M. Nuclear quantum effects enter the mainstream. *Nat. Rev. Chem.* **2018**, *2*, 0109.

(32) Litman, Y.; Donadio, D.; Ceriotti, M.; Rossi, M. Decisive role of nuclear quantum effects on surface mediated water dissociation at finite temperature. *J. Chem. Phys.* **2018**, *148*.

(33) Marx, D. Proton transfer 200 years after von Grotthuss: Insights from ab initio simulations. *ChemPhysChem* **2006**, *7*, 1848–1870.

(34) Komsa, H.-P.; Pasquarello, A. Finite-Size Supercell Correction for Charged Defects at Surfaces and Interfaces. *Phys. Rev. Lett.* **2013**, *110*, 095505.

(35) Zhang, L.; Lin, D.-Y.; Wang, H.; Car, R.; E, W. Active learning of uniformly accurate interatomic potentials for materials simulation. *Phys. Rev. Mater.* **2019**, *3*, 023804.

(36) Zhang, Y.; Wang, H.; Chen, W.; Zeng, J.; Zhang, L.; Wang, H.; E, W. DP-GEN: A concurrent learning platform for the generation of reliable deep learning based potential energy models. *Comput. Phys. Commun.* **2020**, *253*, 107206.

(37) Wang, H.; Zhang, L.; Han, J.; E, W. DeePMD-kit: A deep learning package for many-body potential energy representation and molecular dynamics. *Comput. Phys. Commun.* **2018**, *228*, 178–184.

(38) Thompson, A. P.; Aktulga, H. M.; Berger, R.; Bolintineanu, D. S.; Brown, W. M.; Crozier, P. S.; in 't Veld, P. J.; Kohlmeyer, A.; Moore, S. G.; Nguyen, T. D.; Shan, R.; Stevens, M. J.; Tranchida, J.; Trott, C.; Plimpton, S. J. LAMMPS - a flexible simulation tool for particle-based materials modeling at the atomic, meso, and continuum scales. *Comput. Phys. Commun.* **2022**, *271*, 108171.

(39) Tribello, G. A.; Bonomi, M.; Branduardi, D.; Camilloni, C.; Bussi, G. PLUMED 2: New feathers for an old bird. *Comput. Phys. Commun.* **2014**, *185*, 604–613.

(40) Wiktor, J.; Pasquarello, A. Electron and Hole Polarons at the $BiVO_4$–Water Interface. 2019; `https://doi.org/10.82567/materialscloud:2019.0035/v1`.

(41) Invernizzi, M.; Parrinello, M. Rethinking Metadynamics: From Bias Potentials to Probability Distributions. *J. Phys. Chem. Lett.* **2020**, *11*, 2731–2736.

(42) Invernizzi, M.; Parrinello, M. Exploration vs convergence speed in adaptive-bias enhanced sampling. *J. Chem. Theory Comput.* **2022**, *18*, 3988–3996.

(43) consortium, T. P. Promoting transparency and reproducibility in enhanced molecular simulations. *Nat. Methods* **2019**, *16*, 670–673.

(44) Gao, X.; Chen, J.; Che, H.; Yang, H. B.; Liu, B.; Ao, Y. Accelerating Small Electron Polaron Dissociation and Hole Transfer at Solid–Liquid Interface for Enhanced Heterogeneous Photoreaction. *J. Am. Chem. Soc.* **2024**,

(45) Lyu, S.; Wiktor, J.; Pasquarello, A. Oxygen Evolution at the $BiVO_4$–Water Interface: Mechanism of the Water Dehydrogenation Reaction. *ACS Catal.* **2022**, *12*, 11734–11742.

(46) Lyu, S.; Wiktor, J.; Pasquarello, A. Oxygen evolution at the $BiVO_4$-water interface: mechanism of the water dehydrogenation reaction. 2022; `https://doi.org/10.24435/materialscloud:7w-9v`.

(47) Henkelman, G.; Uberuaga, B. P.; Jónsson, H. A climbing image nudged elastic band method for finding saddle points and minimum energy paths. *J. Chem. Phys.* **2000**, *113*, 9901–9904.

(48) Wiktor, J.; Ambrosio, F.; Pasquarello, A. Role of Polarons in Water Splitting: The Case of $BiVO_4$. *ACS Energy Lett.* **2018**, *3*, 1693–1697.

(49) Ziwritsch, M.; Müller, S.; Hempel, H.; Unold, T.; Abdi, F. F.; Krol, R. v. d.; Friedrich, D.; Eichberger, R. Direct Time-Resolved Observation of Carrier Trapping and Polaron Conductivity in $BiVO_4$. *ACS Energy Lett.* **2016**, *1*, 888–894.

(50) Chen, J.; Li, Y.-F.; Sit, P.; Selloni, A. Chemical Dynamics of the First Proton-Coupled Electron Transfer of Water Oxidation on TiO_2 Anatase. *J. Am. Chem. Soc.* **2013**, *135*, 18774–18777.
